# Sensing Estrogen with Electrochemical Impedance Spectroscopy

**DOI:** 10.1155/2016/9081375

**Published:** 2016-10-10

**Authors:** Jing Li, Byung Kun Kim, Kang-Kyun Wang, Ji-Eun Im, Han Nim Choi, Dong-Hwan Kim, Seong In Cho, Won-Yong Lee, Yong-Rok Kim

**Affiliations:** ^1^Department of Chemistry, Yonsei University, Seoul 120-749, Republic of Korea; ^2^Department of Biosystems and Biomaterials Science and Engineering, Seoul National University, Seoul 151-921, Republic of Korea

## Abstract

This study demonstrates the application feasibility of electrochemical impedance spectroscopy (EIS) in measuring estrogen (17*β*-estradiol) in gas phase. The present biosensor gives a linear response (*R* ^2^ = 0.999) for 17*β*-estradiol vapor concentration from 3.7 ng/L to 3.7 × 10^−4^ ng/L with a limit of detection (3.7 × 10^−4^ ng/L). The results show that the fabricated biosensor demonstrates better detection limit of 17*β*-estradiol in gas phase than the previous report with GC-MS method. This estrogen biosensor has many potential applications for on-site detection of a variety of endocrine disrupting compounds (EDCs) in the gas phase.

## 1. Introduction

While rapid industrialization in the 20th century has introduced a significant degree of convenience to human life, the global population is being exposed to a polluted environment that includes contaminated air such as carbon monoxide, sulfur dioxide, dioxin, formaldehyde, chlorine, bisphenol, and estradiol [1]. Among the pollutants, 4-nonylphenol, bisphenol, and estradiol are classified as endocrine disrupting chemicals (EDCs) and these EDCs are known to be related to various diseases of infertility, spontaneous abortions, birth defects, endometriosis, breast cancer, and so forth [2–8]. Several papers have been published on the detection of EDCs in solution phase using methods such as fluorimetry, NMR spectroscopy, chromatography, enzyme linked receptor assays, and linear sweep voltammetry [9–17]. Recently, a few researchers studied the detection of EDCs using gas chromatography-mass spectrometry and found 4~8 ng/L of EDCs in gas phase [18]. Therefore, the development of EDC detection and measurement technique is in a great demand, particularly, at very low concentration (0.2~141 ng/L of 17*β*-estradiol exists in environment) in gas phase since the extremely low amount of EDCs may cause the fatal diseases to human [19]. Nevertheless, most people are not aware of the seriousness of EDCs in air. Notably, inhalation in an EDC exposed environment is an easy means of being affected by EDCs. Therefore, one of the main challenges in research is to develop a sensitive and reliable method for determination of EDCs in gas phase.

In the present study, a highly sensitive electrochemical biosensor for the detection of the EDC, 17*β*-estradiol, in gas phase has been developed using estrogen receptor-*α* immobilized on a gold electrode. 17*β*-estradiol in gas phase was detected by electrochemical impedance spectroscopy of Fe(CN)_6_^3−^/Fe(CN)_6_^4−^ at the fabricated biosensor. The present biosensor gives a linear response (*R* ^2^ = 0.999) for the 17*β*-estradiol vapor concentration from 3.7 ng/L to 3.7 × 10^−4 ^ng/L. This study is aimed at developing a technique capable of detecting low EDC concentration (>3.7 × 10^−4 ^ng/L) in gas phase.

## 2. Materials 

Estrogen receptor-*α* (ER-*α*, >85.0%) was purchased from Calbiochem (San Diego, USA). Estrogen hormone (17*β*-estradiol, >98%), 3-mercaptopropionic acid (3-MPA, >99%), 3-(3-dimethylaminopropyl)-1-ethylcarbodiimide hydrochloride (EDC, >98%), N-hydroxysuccinimide (NHS, >98%), and bovine serum albumin (BSA, >96%) were purchased from Aldrich. A phosphate buffered saline (PBS) was purchased from Gibco (NY, USA).

**Scheme 1:**
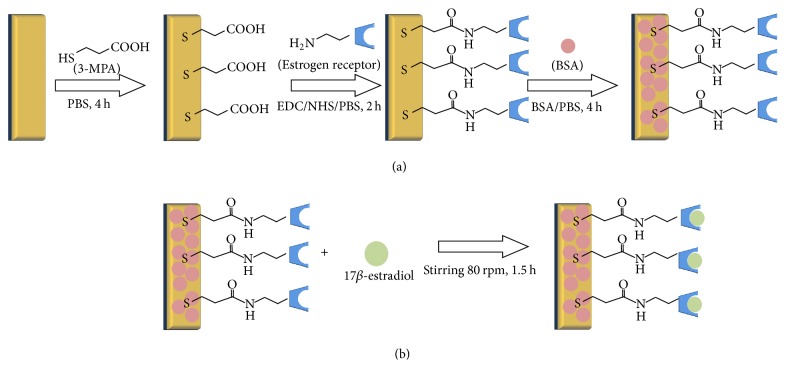
(a) Fabrication of the estrogen biosensor based on the estrogen receptor-*α* modified electrode and (b) the binding of estrogen hormone to the biosensor.

For the gas phase experiment, the estrogen biosensor was prepared as previously reported [20, 21]. The fabricated estrogen biosensor was applied to the detection of 17*β*-estradiol in gas phase. In the experiment, a freshly pretreated gold electrode was incubated in 40 mM 3-MPA solution for 4 h to form a carboxylate terminated self-assembled monolayer (SAM) on the electrode. The electrode was then rinsed using distilled water three times to remove any nonphysically adsorbed 3-MPA molecules before it was stored in distilled water. The 3-MPA modified gold electrode was immersed in a mixture of EDC (1.0 wt.%) and NHS (1.0 wt.%) in 0.05 M PBS (pH 7.4) for 2 h to activate the terminal carboxylic groups, before it was washed using distilled water to remove any nonbonded residual EDC/NHS. Next, a 15 *μ*L aliquot of 0.67 *μ*M estrogen receptor-*α* solution was applied to the surface of the EDC-NHS/3-MPA modified gold electrode. Any nonbonded estrogen receptor-*α* on the EDC-NHS/3-MPA modified gold electrode was removed by rinsing using PBS. Finally, the electrode was incubated in 3.5 wt.% BSA for 4 h to block any nonspecific binding sites. Then the biosensor was dried at 25°C for 4 h and then fixed in the empty headspace of a 10 mL vial which contained only 2 mL of 17*β*-estradiol solution (0.01~100 mM) at the bottom of the vial in order to avoid direct contact between the biosensor and the solution. The vial was stirred at 80 rpm for 90 min (25°C, pH 7). The process was conducted in a sealed vial [22–24]. The schematic diagram of the biosensor fabrication and 17*β*-estradiol binding is illustrated in Scheme 1.

## 3. Methods 

**Figure 1:**
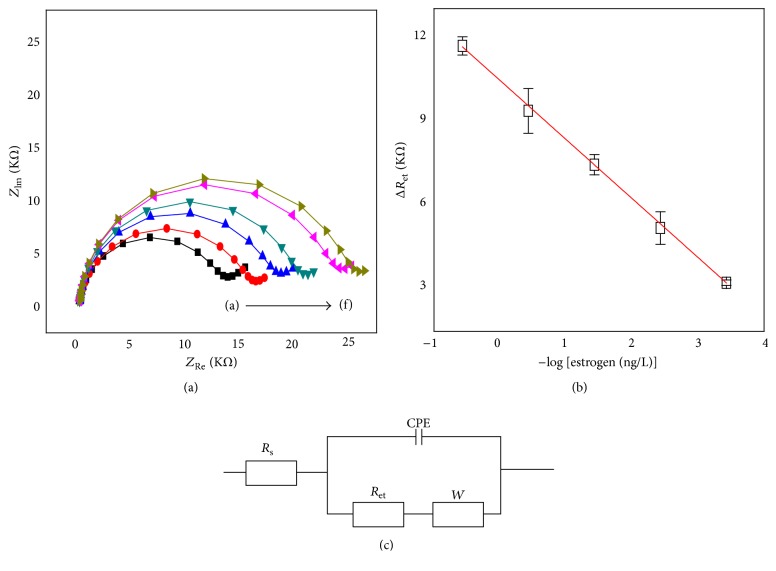
(a) Nyquist plots for the faradaic impedance measurements in the presence of 5.0 mM [Fe(CN)_6_]^3−/4−^ at the estrogen biosensor before (A) and after the treatment of 3.7 × 10^−4^ ng/L (B), 3.7 × 10^−3^ ng/L (C), 3.7 × 10^−2^ ng/L (D), 3.7 × 10^−1^ ng/L (E), and 3.7 ng/L (F) of 17*β*-estradiol in gas phase. (b) Plot of Δ*R*_et_ versus −log[17*β*-estradiol]. *R*_et_(0): electron transfer resistance at the estrogen biosensor before the treatment of 17*β*-estradiol; *R*_et_(*i*): electron transfer resistance at the estrogen biosensor after the treatment of a certain concentration of 17*β*-estradiol. Δ*R*_et_ = *R*_et_(*i*) − *R*_et_(0). (c) Four-component equivalent circuit, *R*_et_: resistance of electron transfer, *R*_*s*_: resistance of solution, *W*: Warburg impedance, and CPE: constant phase element.

Electrochemical impedance experiments were performed using an EG&G 263A potentiostat (Princeton, NJ, USA). A 15 mL electrochemical cell that accommodates a platinum wire counter electrode, the estrogen biosensor as the working electrode, and a Ag/AgCl (3 M NaCl) reference electrode was used. AC impedance spectra were recorded in 0.05 M PBS (pH 7.4) containing 5.0 mM Fe(CN)_6_^3−^ and 5.0 mM Fe(CN)_6_^4−^. The impedance spectra were obtained in the frequency range from 100 MHz to 100 kHz at a bias potential of 0.2 V that was superimposed by an ac potential of 5.0 mV peak-to-peak amplitude. The impedance spectra were plotted in the form of impedance plane plots (Nyquist plots). Zsimpwin program (Princeton Applied Research, Oak Ridge, TN, USA) was used to fit theoretical impedance plots generated based on an equivalent circuit (depicted in Figure 1(c)) to those obtained experimentally. In this way, the diameter of a semicircle in the high frequency range of Nyquist plot was estimated [20, 21].

## 4. Results and Discussion

For the detection of 17*β*-estradiol in gas phase, the estrogen biosensor was exposed to 17*β*-estradiol vapor generated by solutions of concentration between 0.01 and 100 mM for 90 min. Based on Henry's Law, the concentration of 17*β*-estradiol vapor is proportional to that of aqueous 17*β*-estradiol. Henry's Law constant (*k*_H_) of 17*β*-estradiol is known to be 3.64 × 10^−11^ L·atm/mol [25]. SI unit of Henry's Law constant (*k*_H_) was converted to unit of M/atm and the value is calculated to be 2.7 × 10^10^ M/atm, and the corresponding concentration of gaseous 17*β*-estradiol was estimated to be within the range between 0.00037 ng/L and 3.7 ng/L.

As shown in Figure 1(a), the diameter of Nyquist plots, which corresponds to the electron transfer resistance, linearly increases with 17*β*-estradiol concentration. This is because, with increasing 17*β*-estradiol concentration, a corresponding increased quantity of 17*β*-estradiol in the vapor was expected to bind to the immobilized estrogen receptor-*α*, which would in turn gradually hinder the diffusion of [Fe(CN)_6_]^3−^/[Fe(CN)_6_]^4−^ to the biosensor surface. We assume that the 17*β*-estradiol vapor was well bonded to the ER-*α* on the biosensor surface. In order to normalize the data, the impedance change, Δ*R*_et_ = *R*_et_(*i*) − *R*_et_(0), was used in the data analysis, where *R*_et_(*i*) and *R*_et_(0) represent the electron transfer resistance after and before the binding of 17*β*-estradiol to the estrogen biosensor, respectively.

As shown in Figure 1(b), the calibration plot was obtained by graphing the impedance change as a function of the 17*β*-estradiol concentration in gas phase. A linear regression equation was obtained as Δ*R*_et_ = 2195 log⁡[17*β*-estradiol] + 4048 (*R* ^2^ = 0.999, *S*/*N* = 3, *n* = 3). The limit of quantification of the present estrogen biosensor was determined to be 3.7 × 10^−4^ ng/L of 17*β*-estradiol.

The resistance of biosensor was measured with a CPE of electrode since the component has incorporated both the Helmholtz double layer and surface roughness or heterogeneity of the electrode. The values of *R*_*s*_ were fixed at 80 ohm as shown in Table 1. CPE and *W* were determined from the fitting program (ZsimpWin fitting software). The values of *R*_et_ were compensated by the fitting software because the semicircle did not reach to the axis of abscissa in the low frequency region. The fitted values were presented in Table 1.

Association constant (*K*_*a*_) of the binding reaction between 17*β*-estradiol and estrogen receptor-*α* was determined using a Langmuir isotherm approach as follows [26]:

(1) $$\begin{array}{c} \theta =\frac{K_{a}\times C}{1+K_{a}\times C}, \end{array}$$

where *θ* is the fractional coverage of the surface and *C* is the concentration of 17*β*-estradiol vapor. Also, *θ* is expressed by the following equation between *R*_et_(*i*) and *R*_et_(0) [27]:

(2) $$\begin{array}{c} \theta =\frac{R_{et}\left(i\right)-R_{et}\left(0\right)}{R_{et}\left(i\right)}=1-\frac{R_{et}\left(0\right)}{R_{et}\left(i\right)}, \end{array}$$

where *R*_et_(*i*) and *R*_et_(0) represent the electron transfer resistance after and before the binding of 17*β*-estradiol to the estrogen biosensor, respectively. Above equations are further rearranged to

(3) $$\begin{array}{c} \frac{C}{R_{et}\left(i\right)-R_{et}\left(0\right)}=\frac{1}{R_{et}\left(i\right)}C+\frac{1}{K_{a}\times R_{et}\left(i\right)}. \end{array}$$

**Figure 2:**
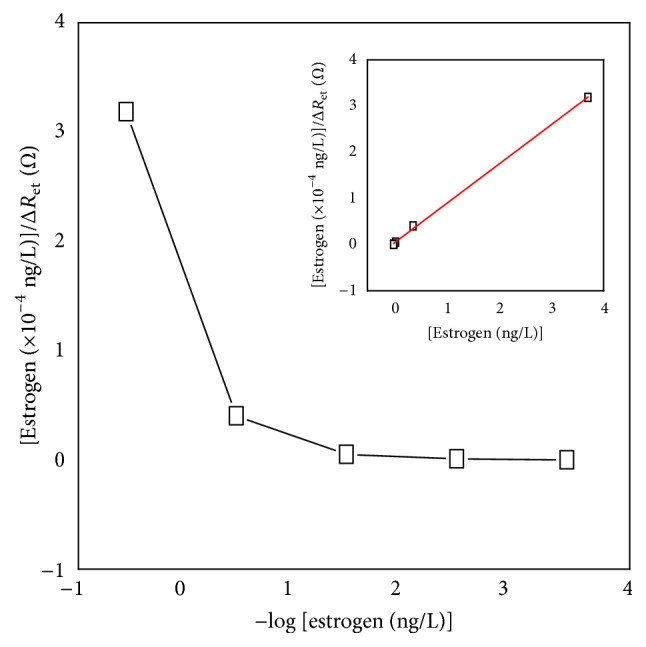
Plot of [17*β*-estradiol]/Δ*R*_et_ versus −log[17*β*-estradiol] in 3.7 × 10^−4^ ng/L to 3.7 ng/L 17*β*-estradiol. Inset: plot of [17*β*-estradiol]/Δ*R*_et_ versus [17*β*-estradiol] in 3.7 × 10^−4^ ng/L to 3.7 ng/L 17*β*-estradiol. Values represent the mean ± standard deviation from three separate experiments. The experiments were repeated by 3 times.

Accordingly, the ordinate intercept of a linear *C*/Δ*R*_et_ versus *C* plot (as shown in Figure 2) will provide an estimate for *K*_*a*_ × *R*_et_(*i*), through which *K*_*a*_ can be evaluated.

Figure 2 reveals that *C*/Δ*R*_et_ linearly changes with the concentration, which results in a linear equation of *C*/Δ*R*_et_ = 8.56 × 10^−5^ *C* + 2.63 × 10^−6^ (*R* ^2^ = 0.999, *N* = 3). From the fitting of the equation, the association constant was observed to be ca. 32.5 L/ng (8.84 × 10^12^ M^−1^). The value of *K*_*a*_ is larger than the reported value of 5.2 × 10^8^ M^−1^ [28]. The result implied that the binding sites on the ER-*α* were well maintained after the introduction of ER-*α* to the surface of modified electrode.

**Figure 3:**
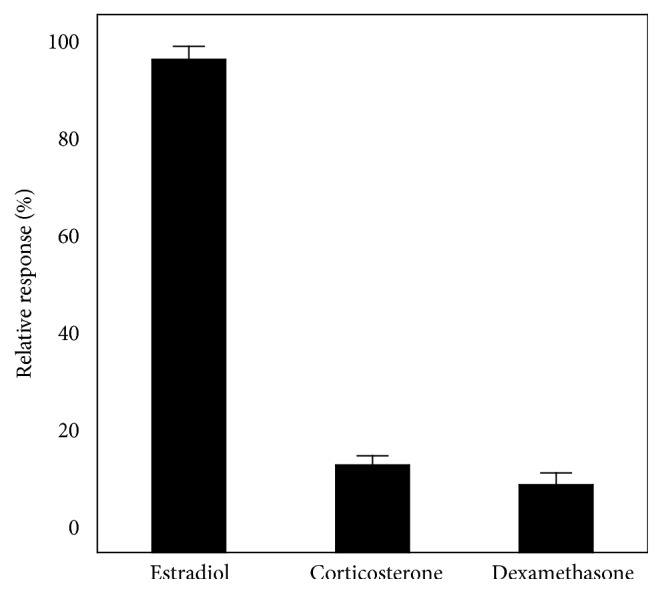
Selectivity test of the estrogen biosensor. Values represent the mean ± standard deviation from three separate experiments.

The identical biosensing measurements were carried out with corticosterone and dexamethasone that have the similar chemical structures without the bonding characteristics to 17*β*-estradiol [29]. The selectivity values were obtained by comparing the impedance values at the high concentrations (3.7 × 10^−1^ ng/L) of corticosterone and dexamethasone with those for 17*β*-estradiol. As shown in Figure 3, the selectivity values for corticosterone and dexamethasone are 9.44 ± 2.2% and 5.05 ± 2.7% due to nonspecific adsorption to the biosensor [30]. The result indicates that the fabricated estrogen biosensor in this study can be used for the selective estimation of 17*β*-estradiol in gas phase.

## 5. Conclusion

In this study, the electrochemical impedance biosensor was successfully developed by immobilizing ER-*α* on a Au electrode and demonstrated for the detection of the gas phase of 17*β*-estradiol. It is considered that such study may open the possibility of the detector development for the detection of EDCs in gas phase.

## Figures and Tables

**Table 1:** Values of the equivalent circuit elements for 17*β*-estradiol detection.

17*β*-estradiol concentration (ng/L)	*R*_*s*_ (Ω)	*Q* (nF)	*R*_et_ (Ω)	*W* (Ω)
0	80	1941	15263	2479
3.7 × 10^−4^	80	2019	16982	3034
3.7 × 10^−3^	80	1917	19890	3219
3.7 × 10^−2^	80	1979	21530	3310
3.7 × 10^−1^	80	1197	25603	3895
3.7	80	2041	26067	3068

## References

[1] Bhatt R. V. (2000). Environmental influence on reproductive health. *International Journal of Gynecology & Obstetrics*.

[2] Liu J. L., Wang R. M., Huang B., Lin C., Wang Y., Pan X. J. (2011). Distribution and bioaccumulation of steroidal and phenolic endocrine disrupting chemicals in wild fish species from Dianchi Lake, China. *Environmental Pollution*.

[3] Granek V., Rishpon J. (2002). Detecting endocrine-disrupting compounds by fast impedance measurements. *Environmental Science and Technology*.

[4] Liehr J. G. (2000). Is estradiol a genotoxic mutagenic carcinogen?. *Endocrine Reviews*.

[5] Wang X., Yang L., Jin X., Zhang L. (2014). Electrochemical determination of estrogenic compound bisphenol F in food packaging using carboxyl functionalized multi-walled carbon nanotubes modified glassy carbon electrode. *Food Chemistry*.

[6] Ragavan K. V., Rastogi N. K., Thakur M. S. (2013). Sensors and biosensors for analysis of bisphenol-A. *TrAC—Trends in Analytical Chemistry*.

[7] Nicolopoulou-Stamati P., Pitsos M. A. (2001). The impact of endocrine disrupters on the female reproductive system. *Human Reproduction Update*.

[8] Hamid H., Eskicioglu C. (2012). Fate of estrogenic hormones in wastewater and sludge treatment: a review of properties and analytical detection techniques in sludge matrix. *Water Research*.

[9] Fan J., Guo H. Q., Liu G. G., Peng P. G. (2007). Simple and sensitive fluorimetric method for determination of environmental hormone bisphenol A based on its inhibitory effect on the redox reaction between peroxyl radical and rhodamine 6G. *Analytica Chimica Acta*.

[10] Jung C., Park J., Lim K. H. (2013). Adsorption of selected endocrine disrupting compounds and pharmaceuticals on activated biochars. *Journal of Hazardous Materials*.

[11] Gómez-Hens A., Aguilar-Caballos M. P. (2003). Social and economic interest in the control of phthalic acid esters. *TrAC—Trends in Analytical Chemistry*.

[12] Matsumoto H., Adachi S., Suzuki Y. (2005). Bisphenol A in ambient air particulates responsible for the proliferation of MCF-7 human breast cancer cells and its concentration changes over 6 months. *Archives of Environmental Contamination and Toxicology*.

[13] Lu J., Wu J., Stoffella P. J., Wilson P. C. (2012). Isotope dilution-gas chromatography/mass spectrometry method for the analysis of alkylphenols, bisphenol A, and estrogens in food crops. *Journal of Chromatography A*.

[14] Hansen M., Krogh K. A., Halling-Sørensen B., Björklund E. (2011). Determination of ten steroid hormones in animal waste manure and agricultural soil using inverse and integrated clean-up pressurized liquid extraction and gas chromatography-tandem mass spectrometry. *Analytical Methods*.

[15] Pieper C., Rotard W. (2011). Investigation on the removal of natural and synthetic estrogens using biofilms in continuous flow biofilm reactors and batch experiments analysed by gas chromatography/mass spectrometry. *Water Research*.

[16] Habauzit D., Boudot A., Kerdivel G., Flouriot G., Pakdel F. (2010). Development and validation of a test for environmental estrogens: checking xeno-estrogen activity by CXCL12 secretion in Breast Cancer Cell Lines (CXCL-test). *Environmental Toxicology*.

[17] Li J. H., Kuang D. Z., Feng Y. L., Zhang F. X., Liu M. Q. (2011). Voltammetric determination of bisphenol A in food package by a glassy carbon electrode modified with carboxylated multi-walled carbon nanotubes. *Microchimica Acta*.

[18] Pérez R. A., Albero B., Tadeo J. L., Molero E., Sánchez-Brunete C. (2014). Analysis of steroid hormones in water using palmitate-coated magnetite nanoparticles solid-phase extraction and gas chromatography-tandem mass spectrometry. *Chromatographia*.

[19] Gomes R. L., Lester J. N., Birkett J. W., Lester J. N. (2002). Endocrine disrupters in drinking water and water reuse. *Endocrine Disruptors in Wastewater and Sludge Treatment Processes*.

[20] Im J.-E., Han J.-A., Kim B. K. (2010). Electrochemical detection of estrogen hormone by immobilized estrogen receptor on Au electrode. *Surface and Coatings Technology*.

[21] Kim B. K., Li J., Im J.-E. (2012). Impedometric estrogen biosensor based on estrogen receptor alpha-immobilized gold electrode. *Journal of Electroanalytical Chemistry*.

[22] Goubaidoulline I., Vidrich G., Johannsmann D. (2005). Organic vapor sensing with ionic liquids entrapped in alumina nanopores on quartz crystal resonators. *Analytical Chemistry*.

[23] Tao S., Xu L., Fanguy J. C. (2006). Optical fiber ammonia sensing probes using reagent immobilized porous silica coating as transducers. *Sensors and Actuators B: Chemical*.

[24] Hämmerle M., Hilgert K., Horn M. A., Moos R. (2011). Analysis of volatile alcohols in apple juices by an electrochemical biosensor measuring in the headspace above the liquid. *Sensors and Actuators B: Chemical*.

[25] Kuster M., José López De Alda M., Barceló D. (2004). Analysis and distribution of estrogens and progestogens in sewage sludge, soils and sediments. *TrAC—Trends in Analytical Chemistry*.

[26] Lyu Y.-K., Lim K.-R., Lee B. Y., Kim K. S., Lee W.-Y. (2008). Microgravimetric lectin biosensor based on signal amplification using carbohydrate-stabilized gold nanoparticles. *Chemical Communications*.

[27] Li P., Ge B., Ou L. M. L., Yao Z., Yu H.-Z. (2015). DNA-redox cation interaction improves the sensitivity of an electrochemical immunosensor for protein detection. *Sensors*.

[28] Hattori K., Takeuchi T., Ogata M. (2007). Detection of environmental chemicals by SPR assay using branched cyclodextrin as sensor ligand. *Journal of Inclusion Phenomena and Macrocyclic Chemistry*.

[29] Rich R. L., Hoth L. R., Geoghegan K. F. (2002). Kinetic analysis of estrogen receptor/ligand interactions. *Proceedings of the National Academy of Sciences of the United States of America*.

[30] Arya S. K., Chornokur G., Venugopal M., Bhansali S. (2010). Dithiobis(succinimidyl propionate) modified gold microarray electrode based electrochemical immunosensor for ultrasensitive detection of cortisol. *Biosensors and Bioelectronics*.

